# Robotic versus Open Thyroidectomy for Differentiated Thyroid Cancer: An Evidence-Based Review

**DOI:** 10.1155/2016/4309087

**Published:** 2016-03-16

**Authors:** Shirley Yuk Wah Liu, Enders Kwok Wai Ng

**Affiliations:** Department of Surgery, Prince of Wales Hospital, Faculty of Medicine, Chinese University of Hong Kong, New Territories, Hong Kong

## Abstract

While open thyroidectomy (OT) is advocated as the gold standard treatment for differentiated thyroid cancer, the contemporary use of robotic thyroidectomy (RT) is often controversial. Although RT combines the unique benefits of the surgical robot and remote access thyroidectomy, its applicability on cancer patients is challenged by the questionable oncological benefits and safety. This review aims to analyze the current literature evidence in comparing RT to OT on thyroid cancers for their perioperative and oncological outcomes. To date, no randomized controlled trial is available in comparing RT to OT. All published studies are nonrandomized or retrospective comparisons. Current data suggests that RT compares less favorably than OT for longer operative time, higher cost, and possibly inferior oncological control with lower number of central lymph nodes retrieved. In terms of morbidity, quality of life outcomes, and short-term recurrence rates, RT and OT are comparable. While conventional OT continues to be appropriate for most thyroid cancers, RT should better be continued by expert surgeons on selected patients who have low-risk thyroid cancers and have high expectations on cosmetic outcomes. Future research should embark on prospective randomized studies for unbiased comparisons. Long-term follow-up studies are also needed to evaluate outcomes on recurrence and survival.

## 1. Introduction

Since the first introduction of endoscopic endocrine neck surgery in 1996 [1, 2], many different techniques of remote access thyroidectomy without a conventional cervical incision have been developed [3, 4]. Although the pursuit on cosmetic superiority can be achieved, remote access thyroidectomy by the endoscopic approach is inevitably associated with the disadvantages of narrow working space, two-dimensional operative view, and restricted instrument manipulation. These have largely limited the applicability of endoscopic thyroidectomy on thyroid neoplasia. Until early 2000s when the Da Vinci robotic system (Intuitive Surgical, Inc., Sunnyvale, CA, USA) was launched, many minimally invasive thyroid surgeons have shifted their enthusiasm towards robotic thyroid surgery [5–10]. The robotic surgical system provides surgeons with the benefits of improved surgical dexterity by multiarticulated instrumentation, stable operative view by hand-tremor filtration technology, and excellent visualization by three-dimensional magnification. These have remarkably extended the indications of the technique onto differentiated thyroid cancers.

Despite the vast abundance of literature reports supporting the safety and effectiveness of robotic thyroidectomy (RT) [11–18], many thyroid surgeons are not convinced on the merits of the procedure in treating thyroid cancers. Substantial controversies over the role of RT have emerged with respect to its safety, perioperative outcomes, and oncological completeness. In October 2011, the Food and Drug Administration had revoked approval for the use of the robot in thyroid surgery in the United States. While excellent outcomes can be confidently achieved by conventional open thyroidectomy (OT) in thyroid cancers, many surgeons who used to be advocates of the robot had stopped practicing RT due to its off-label usage, higher costs, steep learning curve, and unclear patient benefits [19–21]. In this review, we aim to analyze the current literature evidence in comparing RT to OT for the management of differentiated thyroid cancers.

## 2. Robotic Thyroidectomy for Thyroid Cancer

There are three most commonly described RT approaches for thyroid cancers. They are gasless transaxillary approach (TA), bilateral axillobreast approach (BABA), and gasless unilateral axillobreast approach (GUAB). At present, RT is almost exclusively applied on differentiated thyroid cancers (DTC) alone [22–49]. Most reports have no gender restriction but many centers limit their surgery to adults younger than 55 to 70 years [27, 39, 45, 47]. The size limit is commonly set at ≤2–4 cm for low-risk DTC and ≤5 cm for benign or indeterminate nodules [22–49]. RT is not technically feasible for every patient. In terms of disease factor, locally advanced tumors with extrathyroidal invasion to larynx, trachea, esophagus, or recurrent laryngeal nerves, distant metastases, and posteriorly located tumors are considered unsuitable for RT. Minimal anterior strap muscle invasion is often not regarded as a contraindication because en bloc resection of strap muscles can be safely achieved by the robot [26, 29–31]. Presence of multiple metastatic lymph nodes at lateral compartment is no longer a contraindication because concomitant robotic modified radical neck dissection (MRND) can be performed [22, 26, 31]. As for patient factor, prior surgery or irradiation at the neck, breast (for BABA or GUAB), and axilla (for TA or GUAB) are absolute contraindications. Obesity and thyroiditis are relative contraindications in the North American population as a result of perceived operative difficulties [33, 40].

In the literature, more than 25 studies had been published comparing the outcomes between RT and OT for differentiated thyroid cancers [22–49]. Except for three American studies [33, 40, 43], almost all reports are originated from South Korea which represents the main body of worldwide experiences and literature evidences. To date, no randomized controlled trial is available in comparing RT to OT. All available studies are nonrandomized or retrospective comparisons that are subjected to selection biases. In addition to five meta-analyses comparing the outcomes between RT and OT in general [11–15], three more meta-analyses had specifically summarized the differences between RT and OT for thyroid cancer [16–18]. These literature evidences are to be discussed here with respect to the comparisons on the perioperative and oncological outcomes.

### 2.1. Perioperative Outcomes

#### 2.1.1. Operative Time

As consistently shown in 19 comparative studies [22–35, 38, 40, 41, 43–47] and 7 meta-analyses [11–17] comparing RT to OT, the operative time of RT was significantly longer than that of OT for pooled mean differences of 39–54 minutes irrespective of the operative approaches. Although the operative time of RT could be reduced with accumulation of experiences, it was hardly possible for RT to achieve comparable operative time with that of OT [50–52]. This was mainly attributed to the need of extra time for skin flap dissection and robotic docking.

#### 2.1.2. Hospital Stay

The length of hospital stay was analyzed in 15 comparative studies [22–34, 40, 41] and 4 meta-analyses [13–16]. Except for three studies [26, 30, 33], all available data did not find a significant difference between RT and OT for the length of hospital stay.

#### 2.1.3. Postoperative Pain

In addition to pain in the neck, pain at remote wound sites and skin flaps are inherently associated with RT. Using different assessment scales, postoperative pain had been compared in 8 studies [22–24, 27, 28, 40, 44, 48]. Except for two studies reporting significantly less pain in RT than in OT on the first two postoperative days [23, 44], no difference was observed between RT and OT for postoperative pain scores [27, 40, 48] and analgesic requirement [23, 27, 40] in all studies. Chronic pain after 3–6 months was also similar between the two procedures [22, 24, 28]. Due to the adoption of different measurement scales, meta-analytical comparison of postoperative pain from these studies was difficult. Overall evidence suggested that RT and OT were comparable for postoperative pain.

#### 2.1.4. Recurrent Laryngeal Nerve Injury

In the literature, there was no universal consensus in defining transient and permanent recurrent laryngeal nerve (RLN) injuries. Their quoted incidences in RT varied between 1–7% for transient injury and 0–2% for permanent injury (Table 1). Despite the discrepancies in their definitions, no significant difference was observed between RT and OT for the rates of transient and permanent RLN injuries in 18 studies [22–39]. Except for one meta-analysis by Lang et al. [11], all 6 available meta-analyses consistently found no difference between RT and OT for the rates of transient and permanent RLN injuries [12–17]. The current evidence still supported that the risk of RLN injuries was not increased by RT.

#### 2.1.5. Hypoparathyroidism

The definitions of postoperative transient and permanent hypoparathyroidism also varied among different studies (Table 2). The reported rates of transient and permanent hypoparathyroidism in RT ranged from 0–53% and 0–3%, respectively. In 18 studies comparing RT to OT, all except three studies [24, 30, 38] reported no significant difference between the two groups for the rates of transient hypoparathyroidism while the rates of permanent hypoparathyroidism were comparable among all studies [22–38, 40]. Despite the slight variation in the definitions, all available meta-analyses consistently showed that the rates of permanent hypoparathyroidism were comparable between RT and OT [11–17]. As for transient hypoparathyroidism, except for the studies by Jackson et al. and Kandil et al. [12, 15], the pooled results from all other meta-analyses revealed no significant difference between RT and OT [11, 13, 14, 16, 17]. Hence, the current evidence also supported that the risk of hypoparathyroidism was not increased by RT.

#### 2.1.6. Bleeding and Hematoma

Although bleeding and hematoma are the major lethal complications of thyroidectomy, the reported incidences in RT remained below 2-3% and were comparable with those of OT in 16 studies [22–33, 35, 37, 38, 40]. As reflected by 6 meta-analyses, the rates of bleeding and hematoma were similar between RT and OT [11–15, 17].

#### 2.1.7. Other Complications

According to the results from the latest meta-analyses, the outcomes of RT and OT were comparable for the rates of seroma formation [12–15, 17], chyle leak [12–17], and tracheal injury [15]. One study also reported a significantly lower degree of postoperative nausea and vomiting in RT [44]. Wound infection rates were similar between RT and OT in 3 other studies [22, 23, 29].

#### 2.1.8. Voice Dysfunction

Postoperative voice change independent of RLN injury had been compared in 5 studies [22, 27, 39, 45, 47]. Using subjective symptom questionnaires, Tae et al. found that the subjective postoperative voice functions at 1 day, 1 month, and 3 months were significantly better in RT (GUAB) than in OT [45]. When they extended their study duration to 2-year follow-up, RT was found to have advantages of better recovery of voice symptoms and acoustic parameters over OT [47]. In three other studies using TA in RT [22, 27, 39], subjective and objective voice dysfunctions were contrarily found to be comparable between RT and OT. More prospective studies are needed before convincing conclusion can be drawn on the differences in voice dysfunction between RT and OT.

#### 2.1.9. Swallowing Dysfunction

In three studies evaluating the subjective swallowing dysfunction, RT was shown to have significantly less dysfunction than OT in two studies [22, 27] but was similar to OT in another study [45]. More prospective evidence is needed to truly compare such outcome between the two procedures.

#### 2.1.10. Sensory Change

Due to more extensive skin flap dissection, chest paresthesia was significantly more common after RT than OT in both TA and BABA techniques [22, 27, 38]. In a prospective study by Kim et al, anterior chest paresthesia after BABA thyroidectomy was found to be completely normalized by 3 months [53]. While Lee et al. reported significantly more neck paresthesia in OT than in RT using TA [22, 27], Song et al. found no such difference in OT and RT using BABA [38].

#### 2.1.11. Cosmetic Satisfaction

Cosmetic superiority was considered to be the most concerned advantage of RT. In all 6 studies comparing cosmetic satisfaction between RT and OT at different time-points from postoperative 1 day to 6 months, RT was associated with significantly higher cosmetic satisfaction scores irrespective of the operative approaches [22, 24, 27, 28, 32, 34]. In a prospective study evaluating the postoperative body image by Lee et al, significantly better self-body image scores were found in RT than in OT from postoperative 3 months till 9 months [34]. In another study analyzing the postoperative cosmetic concerns by Koo do et al., the degree of scarring and psychological distress were also significantly better after RT than OT [48]. Hence, the cosmetic benefits of RT were confirmed by the current literature evidences.

#### 2.1.12. Cost

One of the greatest disadvantages of RT is cost. Based on a cost model consisting of operating room charges, anesthesia fee, consumable cost, equipment depreciation, and maintenance cost, Cabot et al. found that RT was 1.5 times more expensive than OT (USD 13670 ± 1384 versus 9028 ± 891, *P* < 0.001) [54]. Even when the annual case load was increased to reduce the equipment depreciation cost per case, such cost difference between RT and OT could hardly be resolved. In another study analyzing the relative costs, Broome et al. also found that the cost of RT was 2.1 times higher than that of OT (USD 5797 versus 2668) [55]. In two recent comparative studies on thyroid cancer, the cost of RT was again shown to be significantly higher than that of OT [31, 32].

### 2.2. Oncological Outcomes

#### 2.2.1. Lymph Node Retrieval

Radical central compartment nodal dissection (CCD) often represents the hallmark of favorable oncological control. In 15 studies comparing the number of LN retrieved during CCD [22–32, 35–37, 41], 6 reported a significantly lower number of LN retrieved in RT than in OT but 9 other studies found comparable results between the two procedures (Table 3). In three meta-analyses, the number of central LN retrieved was consistently lower in RT than in OT though the reported absolute differences were small (number <1) [16–18]. As for the number of LN retrieved during MRND for more advanced disease stages, only three studies compared RT to OT and no significant difference was found [22, 26, 31]. Based on the evidence from pooled analyses, RT might be inferior to OT in terms of the number of central LN retrieved.

#### 2.2.2. Surgical Completeness of Resection

In thyroid cancer, surgical completeness of resection is commonly estimated by the serum thyroglobulin (Tg) levels and the RAI uptake levels on posttherapy whole-body scan (RxWBS) at radioiodine (RAI) ablation. The thyrotropin- (TSH-) stimulated Tg (sTg) level is a reliable surrogate marker for the amount of remnant thyroid tissue after total thyroidectomy. It is measured upon TSH stimulation by either thyroid hormone withdrawal or human recombinant TSH stimulation. Ablation sTg is measured at the time of RAI ablation while control sTg is measured at 6–12 months after RAI. In the literature, ablation sTg levels were reported to be significantly higher in RT than in OT in 4 studies [24, 30, 34, 35] but were similar between the two groups in 5 other studies [29, 31, 36, 37, 42]. Although two meta-analyses by Wang et al. and Son et al. found no difference between RT and OT for sTg levels [16, 17], another meta-analysis consisting of the highest number of individual studies (*n* = 6) by Lang et al. contrarily revealed a significantly higher sTg level in RT than in OT [18]. As for control sTg, its levels were compared in three studies and no significant difference was observed between RT and OT [24, 30, 35]. Overall evidence suggested that the amount of remnant thyroid tissue (as reflected by ablation sTg levels) might be higher in RT than in OT but such difference was resolved after RAI ablation. Using abnormal RAI uptake at RxWBS to represent the amount of remnant thyroid tissues, 6 studies reported comparable results between RT and OT [25, 26, 34, 35, 40, 42] but one study found a significantly higher result in RT [36]. The available evidence was considered inadequate for drawing a definite conclusion on the surgical completeness of resection by the two procedures.

#### 2.2.3. Tumor Recurrence

Short-term locoregional recurrence within the first two postoperative years was comparable between RT and OT in 7 studies [22–26, 35, 40]. In the only long-term follow-up study by Lee et al, the rates of locoregional recurrence at 5 years were similar between RT and OT (1.2% versus 1.2%) [41].

#### 2.2.4. Survival Outcomes

In the literature, the survival outcomes between RT and OT were only compared in one study [41]. At 5-year follow-up, Lee et al. observed no significant difference between RT and OT for the disease-free survival (99.7% versus 98.7%, *P* = 0.89). Longer time is needed to wait for more long-term follow-up data before comparison on survival outcomes can be made.

### 2.3. Generalizability of Evidence

As stated before, almost all the available evidences about the use of RT on thyroid cancer were originated from South Korea. The generalizability of these results to the North American or European populations is questionable because of the differences in body habitus, incidence of subcentimeter nodules, prevalence of obesity, and occurrence of thyroiditis in different ethnic groups [10].

## 3. Conclusion

Despite the established advantages on cosmesis, current data suggests that RT compares less favorably than OT for longer operative time, higher cost, and possibly inferior oncological control with lower number of central LN retrieved. In terms of morbidity and quality of life outcomes, RT and OT are comparable for thyroid cancer patients. While conventional OT continues to be appropriate for most thyroid cancer patients, RT should better be continued by expert surgeons on selected patients who have low-risk thyroid cancers and have high expectations on cosmetic outcomes. More prospective long-term follow-up studies are needed to define the oncological safety of RT.

## Figures and Tables

**Table 1:** Summary of recurrent laryngeal nerve injury in published studies.

First author/year	RT approach	Sample size (RT versus OT)	Definition of transient RLN injury	Rates of transient RLN injury (%) (RT versus OT)	Definition of permanent RLN injury	Rates of permanent RLN injury (%) (RT versus OT)
Lee, 2013 [22]	TA	62 : 66	Not stated	3.2 : 4.5 (*P* = 0.21)	Not stated	0 : 0 (*P* = NS)

Ryu, 2013 [23]	TA	45 : 45	—	NR	Not stated	0 : 0 (*P* = NS)

Tae, 2012 [24]	GUAB	75 : 226	Vocal cord palsy on laryngoscopy with recovery within 6 months	8.0 : 3.1 (*P* = 0.09)	Vocal cord palsy on laryngoscopy failed to recover after 6 months	0 : 0.4 (*P* = 1.0)

Lee, 2012 [25]	TA	192 : 266	Not stated	2.6 : 0.4 (*P* = 0.08)	Not stated	6.8 : 0 (*P* = 0.07)

Kang, 2012 [26]	TA	56 : 109	Not stated	3.6 : 2.8 (*P* = 0.77)	Not stated	0 : 0 (*P* = NS)

Lee, 2010 [27]	TA	41 : 43	Vocal cord palsy on laryngoscopy with recovery within 6 months	2.4 : 0 (*P* = NS)	Vocal cord palsy on laryngoscopy not recovered after 6 months	0 : 0 (*P* = NS)

Tae, 2011 [28]	GUAB	41 : 163	Not stated	2.4 : 2.5 (*P* = 0.73)	Not stated	0 : 0.6 (*P* = 0.79)

Kim, 2011 [29]	BABA	69 : 138	Vocal cord palsy on laryngoscopy not recovering within 6 months	1.4 : 0.7 (*P* = 0.62)	Failure of voice change to normalize after 6 months	0 : 0 (*P* = NS)

Yi, 2013 [30]	TA	98 : 423	Not stated	1.0 : 0.5 (*P* = 0.46)	—	NR

Kim, 2015 [31]	BABA	13 : 65	Vocal cord palsy on laryngoscopy lasting for <6 months	0 : 4.6 (*P* = NS)	Vocal cord palsy on laryngoscopy lasting for >6 months	0 : 3.1 (*P* = NS)

Kwak, 2015 [32]	BABA	206 : 634	Vocal cord palsy on stroboscopy from 2 weeks to 6 months	0.9 : 0.5 (*P* = 0.36)	—	NR

Noureldine, 2013 [33]	TA	24 : 35	Vocal cord palsy on laryngoscopy lasting for <6 months	4.1 : 5.7 (*P* = 0.76)	Vocal cord palsy on laryngoscopy persisting after 6 months	0 : 0 (*P* = NS)

Lee, 2014 [34]	TA	60 : 56	Not stated	5.0 : 0 (*P* = 0.24)	—	NR

Tae, 2014 [35]	GUAB	62 : 183	Not stated	6.5 : 2.2 (*P* = 0.11)	Not stated	0 : 0 (*P* = NS)

Lee, 2014 [36]	TA	43 : 51	Not stated	2.3 : 0 (*P* = 0.45)	—	NR

Kim, 2014 [37]	BABA	123 : 392	Vocal cord palsy persisted <6 months	4.9 : 6.1 (*P* = 0.60)	Vocal cord palsy persisted >6 months	0 : 0.3 (*P* = 1.0)

Song, 2014 [38]	GUAB	118 : 176	Not stated	0.8 : 2.8 (*P* = 0.40)	Not stated	0 : 0 (*P* = NS)

Lee, 2012 [39]	TA	42 : 46	Vocal fold motion impairment on videolaryngostroboscopy from 1 week to 3 months	21.4 : 19.5 (*P* = 0.82)	—	NR

RT, robotic thyroidectomy; OT, open thyroidectomy; RLN, recurrent laryngeal nerve; TA, transaxillary approach; GUAB, gasless unilateral axillobreast approach; BABA, bilateral axillobreast approach; *P*, *P* value; NS, nonsignificant; NR, not reported.

**Table 2:** Summary of hypoparathyroidism in published studies.

First author/year	RT approach	Sample size (RT versus OT)	Definition of transient hypoparathyroidism	Rates of transient hypoparathyroidism (%) (RT versus OT)	Definition of permanent hypoparathyroidism	Rates of permanent hypoparathyroidism (%) (RT versus OT)
Lee, 2013 [22]	TA	62 : 66	Drop in PTH with recovery <6 months	38.7 : 34.8 (*P* = 0.56)	Drop in PTH with no recovery after 6 months	0 : 0 (*P* = NS)

Ryu, 2013 [23]	TA	45 : 45	—	NR	Not stated	0 : 0 (*P* = NS)

Tae, 2012 [24]	GUAB	75 : 226	PTH below normal limit but recovered within 6 months	27.5 : 49.5 (*P* < 0.001)	PTH below normal limit and persisted for >6 months	0 : 1.8 (*P* = 0.57)

Lee, 2012 [25]	TA	192 : 266	Not stated	44.4 : 40.0 (*P* = 0.82)	Not stated	0 : 3.0 (*P* = 0.58)

Kang, 2012 [26]	TA	56 : 109	Not stated	48.2 : 45.9 (*P* = 0.77)	Not stated	0 : 0 (*P* = NS)

Lee, 2010 [27]	TA	41 : 43	Not stated	19.2 : 15.3 (*P* = NS)	Not stated	0 : 0 (*P* = NS)

Tae, 2011 [28]	GUAB	41 : 163	Not stated	20.0 : 30.1 (*P* = 0.39)	Not stated	0 : 4.2 (*P* = 0.65)

Aliyev, 2013 [40]	TA	16 : 30	Serum Ca <8 mg/dL for ≤2 weeks	12 : 13 (*P* = NS)	—	NR

Kim, 2011 [29]	BABA	69 : 138	Normalization of PTH within 6 months	33.3 : 27.5 (*P* = 0.58)	Failure of normalization of PTH after 6 months	1.4 : 2.9 (*P* = NS)

Yi, 2013 [30]	TA	98 : 423	Symptomatic and/or serum Ca <7.5 mg/dL for ≤6 months	53.1 : 43.0 (*P* = 0.04)	Symptomatic and/or serum Ca <7.5 mg/dL for >6 months	3.1 : 0.7 (*P* = 0.08)

Kim, 2015 [31]	BABA	13 : 65	Serum ionized Ca <4.0 mEq/L or symptoms requiring Ca replacement	0 : 15.4 (*P* = NS)	Not stated	0 : 1.5 (*P* = NS)

Kwak, 2015 [32]	BABA	206 : 634	Serum ionized Ca <4.4 mg/dL or PTH <8 pg/mL within 1 year	15 : 14.6 (*P* = 0.29)	Serum ionized Ca <4.4 mg/dL or PTH <8 pg/mL persisted after 1 year and need of Ca supplement	0.3 : 0.5 (*P* = 0.08)

Noureldine, 2013 [33]	TA	24 : 35	Abnormal Ca level persisted <6 months	8.3 : 11.4 (*P* = 0.66)	Abnormal Ca level persisted >6 months	0 : 0 (*P* = NS)

Lee, 2014 [34]	TA	60 : 56	Not stated	42 : 45 (*P* = 0.85)	—	NR

Tae, 2014 [35]	GUAB	62 : 183	Not stated	43.5 : 37.1 (*P* = 0.45)	Not stated	1.6 : 1.6 (*P* = 0.73)

Lee, 2014 [36]	TA	43 : 51	Not stated	46.5 : 31.4 (*P* = 0.14)	—	NR

Kim, 2014 [37]	BABA	123 : 392	Low PTH within 6 months	23.4 : 22.0 (*P* = 0.16)	Low PTH persisted for >6 months	0 : 0 (*P* = NS)

Song, 2014 [38]	GUAB	118 : 176	Not stated	35.7 : 55.9 (*P* = 0.006)	Not stated	0 : 0.7 (*P* = 0.67)

RT, robotic thyroidectomy; OT, open thyroidectomy; TA, transaxillary approach; GUAB, gasless unilateral axillobreast approach; BABA, bilateral axillobreast approach; PTH, parathyroid hormone; Ca, calcium; *P*, *P* value; NS, nonsignificant; NR, not reported.

**Table 3:** Summary of oncological outcomes in published studies.

First author/year	RT approach	Sample size (RT versus OT)	Mean number of central LN retrieved (RT versus OT)	Mean ablation sTg (ng/mL) (RT versus OT)
Lee, 2013 [22]	TA	62 : 66	8.1 : 7.9 (*P* = 0.21)	NR

Ryu, 2013 [23]	TA	45 : 45	5.7 : 7.0 (*P* = 0.23)	NR

Tae, 2012 [24]	GUAB	75 : 226	4.4 : 7.7 (*P* < 0.001)	12.7 : 4.9 (*P* = 0.03)

Lee, 2012 [25]	TA	192 : 266	4.6 : 5.7 (*P* = 0.004)	NR

Kang, 2012 [26]	TA	56 : 109	6.5 : 8.6 (*P* = 0.03)	NR

Lee, 2010 [27]	TA	41 : 43	4.4 : 4.3 (*P* = 0.84)	NR

Tae, 2011 [28]	GUAB	41 : 163	4.7 : 9.6 (*P* < 0.01)	NR

Kim, 2011 [29]	BABA	69 : 138	4.7 : 4.8 (*P* = 0.80)	0.8 : 0.8 (*P* = 0.97)

Yi, 2013 [30]	TA	98 : 423	6.5 : 7.0^∧^ (*P* = 0.57)	26% : 10.6%^*∗*^ (*P* = 0.001)

Kim, 2015 [31]	BABA	13 : 65	12.8 : 12.7 (*P* = 0.97)	2.5 : 2.8 (*P* = NS)

Kwak, 2015 [32]	BABA	206 : 634	5.8 : 8.4 (*P* = 0.001)	NR

Lee, 2014 [34]	TA	60 : 56	NR	5.3 : 1.6 (*P* = 0.005)

Tae, 2014 [35]	GUAB	62 : 183	4.1 : 5.4 (*P* = 0.24)	10.2 : 3.8 (*P* < 0.001)

Lee, 2014 [36]	TA	43 : 51	4.9 : 6.3 (*P* = 0.06)	4.4 : 4.1 (*P* = 0.67)

Kim, 2014 [37]	BABA	123 : 392	8.7 : 10.4 (*P* = 0.006)	1.3 : 1.1 (*P* = 0.65)

Lee, 2015 [41]	TA	206 : 206	5.8 : 6.6 (*P* = 0.10)	NR

Lee, 2011 [42]	BABA	174 : 237	NR	1.4 : 1.2 (*P* = 0.99)

RT, robotic thyroidectomy; OT, open thyroidectomy; LN, lymph nodes; sTg, thyrotropin-stimulated serum thyroglobulin; TA, transaxillary approach; GUAB, gasless unilateral axillobreast approach; BABA, bilateral axillobreast approach; *P*, *P* value; NS, nonsignificant; NR, not reported. ^∧^Median (range). ^*∗*^Percentage of patients with abnormal ablation sTg (level >2 ng/mL).
